# Inverse association between type 2 diabetes and hepatocellular carcinoma in East Asian populations

**DOI:** 10.3389/fendo.2023.1308561

**Published:** 2024-01-03

**Authors:** Jinlong Huo, Yaxuan Xu, Xingqi Chen, Jie Yu, Lijin Zhao

**Affiliations:** ^1^ Department of General Surgery, Digestive Disease Hospital, Affiliated Hospital of Zunyi Medical University, Zunyi, Guizhou, China; ^2^ Department of Breast and Thyroid Surgery, The Third Affiliated Hospital of Zunyi Medical University (The First People’s Hospital of Zunyi), Zunyi, Guizhou, China

**Keywords:** type 2 diabetes, hepatocellular carcinoma, Mendelian randomization, East Asian populations, association

## Abstract

**Aims:**

To investigate the potential association between type 2 diabetes (T2D) and hepatocellular carcinoma (HCC) in East Asian populations using Mendelian randomization (MR) analyses.

**Methods:**

Bidirectional Mendelian randomization (MR) studies were conducted using summary statistics from genome-wide association studies (GWAS) related to T2D and HCC. The potential effects of confounders such as chronic hepatitis B, chronic hepatitis C, body mass index, and alcohol intake frequency were corrected using a multivariate MR study. Various MR methods, including the inverse variance weighted (IVW) approach, were used to estimate the associations between T2D and HCC. Sensitivity analysis and assessment of heterogeneity were performed to ensure the robustness of the results.

**Results:**

In the forward MR study, the IVW approach of MR analysis suggested an inverse association between T2D and HCC, with a risk odds ratio of 0.8628 (95% confidence interval [CI], 0.7888–0.9438). Furthermore, even after adjusting for BMI, chronic hepatitis B, and alcohol intake frequency, this study still supports the inverse association between T2D and HCC. Additional MR methods provided further support for this relationship. Sensitivity analysis and assessment of heterogeneity confirmed the robustness of the results. The reverse MR analysis did not show a clear impact of genetic liability to HCC on reduced risk of T2D(OR=0.9788; 95% CI, 0.9061-1.0574).

**Conclusion:**

This study provides evidence of an inverse association between T2D and HCC in East Asian populations using MR analysis. Further studies are warranted to validate these findings.

## 1 Introduction

Hepatocellular carcinoma (HCC) is the predominant subtype of liver cancer and poses an alarming health challenge. Research estimates indicate that HCC makes up about 75-85% of all liver cancer cases worldwide (1). HCC incidence has exhibited a steady rise over the past few decades, thus intensifying the gravity of public health implications. Type 2 diabetes (T2D) is a metabolic disorder defined by hyperglycemia and insulin resistance (2, 3). It is a significant global health issue affecting millions of individuals worldwide. The incidence of T2D has been persistently increasing and is projected to continue to rise in the foreseeable future (4, 5).

The relationship between T2D and HCC is still a matter of debate. Chronic hepatitis B virus (HBV) and hepatitis C virus (HCV) infections, along with obesity and alcohol consumption, have been widely established as significant risk factors for the development of HCC (6–9). Moreover, chronic hyperglycemia, insulin resistance, and inflammation, common in T2D, are also believed to contribute to HCC development and progression (10, 11). Although T2D is suggested to be the most important risk factor for HCC (12), considering the possibility of reverse causality, the question of whether diabetes increases the risk of HCC independently of other risk factors has not been definitively answered.

Based on current research, both chronic HCV itself and HCV-associated cirrhosis may increase the risk of subsequent development of T2D, and existing diabetes appears to accelerate the progression of chronic HCV infection to cirrhosis (13, 14). Similarly, hepatitis B is also more prevalent in individuals with T2D, with a higher risk of chronic infection and liver-related complications (15). Excessive alcohol consumption is a well-established risk factor for the development of HCC. In patients with T2D, excessive alcohol consumption has been associated with an increased risk of liver complications and metabolic syndrome (16).Shared risk factors, such as obesity, alcohol consumption, hepatitis B or C infections and other risk factors, further complicate the T2D-HCC relationship (17, 18). Therefore, establishing a causal link remains challenging due to confounding factors and reverse causality.

Mendelian randomization (MR) employs genetic variants as instrumental variables to investigate the causal link between the exposure (such as T2D) and the outcome (such as HCC) (19, 20). This approach can overcome limitations of traditional observational studies, such as confounding and reverse causality. Two types of MR approaches, namely bidirectional MR and multivariable MR, have gained attention for their ability to address confounding factors (21). Bidirectional MR investigates the association between the exposure and an outcome by using genetic variants associated with the exposure as instrumental variables and genetic variants associated with the outcome as instrumental variables (22). This approach helps to explore whether the exposure is a risk factor for outcome or vice versa. Multivariable MR, on the other hand, allows for the adjustment of multiple confounding factors simultaneously (23). By incorporating genetic variants associated with potential confounders, such as obesity or viral hepatitis infections, this approach helps to remove the influence of these factors on the relationship between the exposure and outcome, providing more robust evidence.

Given the high prevalence of T2D and HCC in East Asian populations, understanding the association between these two conditions is crucial for developing effective prevention and treatment strategies.By employing bidirectional MR and multivariable MR, we aims to elucidate the potential causal association between T2D and HCC risk, while also accounting for confounding factors. The findings of this study will contribute to our understanding of the aetiology of HCC in East Asian populations and may have implications for the development of targeted interventions to reduce the burden of T2D and HCC in these populations.

## 2 Materials and methods

### 2.1 Study design

**Figure 1:**
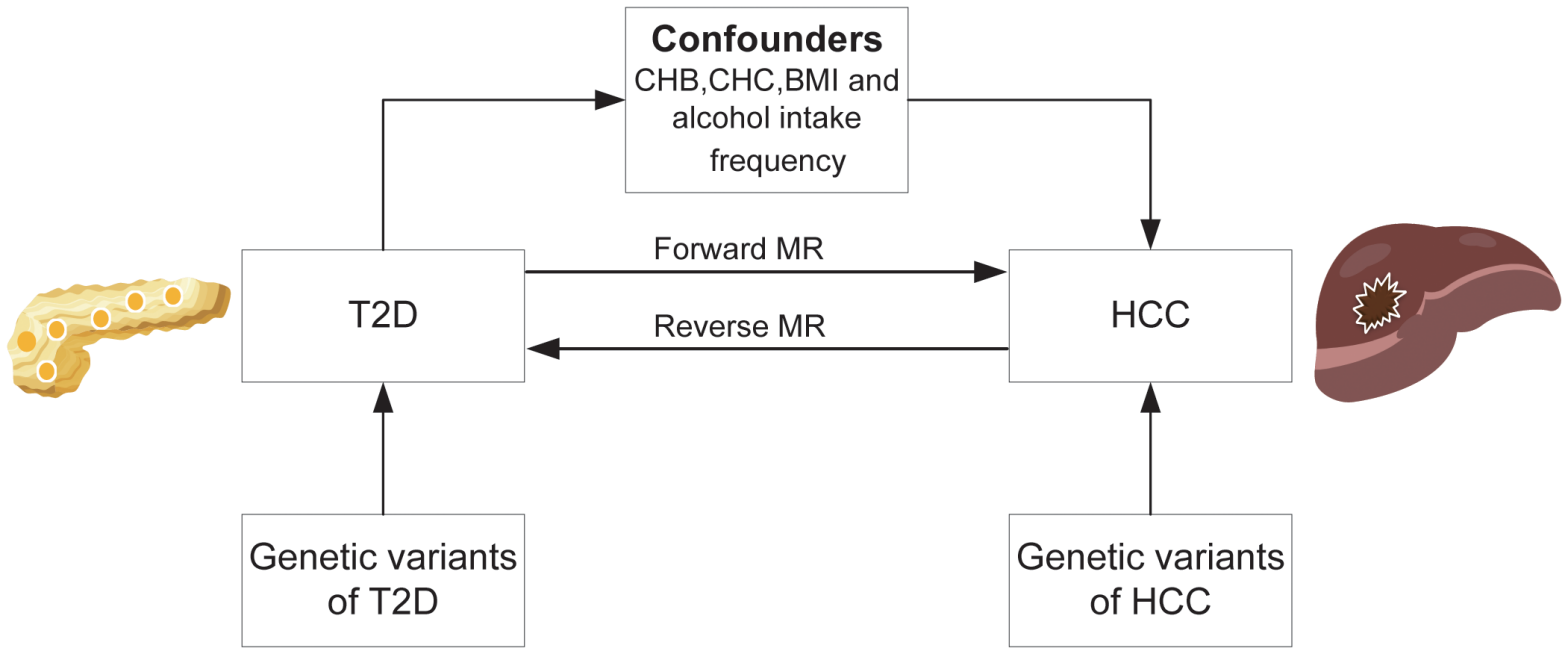
Overall study design based on Mendelian randomization analysis. T2D, type 2 diabetes; HCC, hepatocellular carcinoma; CHB; chronic hepatitis B; CHC, chronic hepatitis C; BMI, body mass index.

See **Figure 1** for the study design. In the MR study, summary-level data of genome-wide association analyses on T2D, HCC, and confounding factors including Chronic hepatitis B(CHB), Chronic hepatitis C(CHC), body mass index(BMI), and alcohol intake frequency from published genome-wide association studies (GWASs). We first performed a forward MR study to examine the causative risk of T2D for HCC and corrected for confounders including CHB, CHC, BMI and alcohol intake frequency using multivariable MR analysis. We then performed reverse MR analysis to investigate the relationship between genetic susceptibility to HCC and T2D. The GWAS cited in this article were all approved by the relevant review board. The MR study did not need an ethical permit based on summary-level data.

### 2.2 Data sources for T2D

One hundred and seventy-four single-nucleotide polymorphisms (SNPs) associated with T2D were identified at the genome-wide significance threshold (*p*<5×10^-8^) from a meta analysis of recently published GWASs including 433,540 individuals of East Asian descent(77,418 cases and 356,122 controls) (24). Sixteen palindromic SNPs (rs10830963, rs11257657, rs256904, rs340875, rs4237150, rs4736999, rs4739515, rs475002, rs6885132, rs7090695, rs7210161, rs7900112, rs8037894, rs838720, rs9368194, rs988748) with intermediate allele frequencies was removed. Finally, one hundred and fifty-eight SNPs without linkage were chosen as instrumental variables for T2D (r^2^<0.001, window size =10,000 kb). (**Supplementary Table 1**) Association analyses were adjusted for gender and body mass index(BMI). These instruments have also been used in previous MR studies (25, 26). Additionally,We also used this summary-level data for T2D in the reverse MR analysis.

### 2.3 Data sources for HCC

Summary-level data for the associations of T2D associated SNPs with HCC were obtained from a meta-analysis of GWAS from Biobank Japan (1,866 cases and 195,745 controls of East Asian descent) (27, 28). Cases in Biobank Japan were defined by International Classification of Disease code ICD10 C. For the reverse MR analysis, three independent SNPs(rs1131500, rs113777417, rs8107030) associated with HCC at the genome-wide significance threshold (*P* < 5×10^–8^) were selected as instrumental variables from the Sakaue et al. GWAS(r2<0.01). (**Supplementary Table 2**) (28) The three SNPs had no significant association with T2D and were used as instrumental variables in the reverse sensitivity analysis.

### 2.4 Data sources for confounding factors

Four confounding factors, including Chronic hepatitis B(CHB), Chronic hepatitis C(CHC), body mass index(BMI) and alcohol intake frequency, possibly associated with HCC were selected (29). Summary data on these confounding factors are from the second wave of genome-wide association analyses from IEU-OpenGWAS project (https://gwas.mrcieu.ac.uk/), with GWAS ID:bbj-a-99, bbj-a-101, bbj-a-1,and ukb-e-1558_EAS, respectively. To mitigate the effects of racial differences, the populations in these GWAS data related to confounders were all of East Asian ancestry.

### 2.5 Statistical analysis

Bidirectional MR was employed to investigate the direction of causation between T2D and HCC. In forward MR analysis, The inverse-variance weighted (IVW) was used as the primary method to estimate the associations between exposure (T2D) and outcome (HCC). Risk estimates are expressed as odds ratios (ORs) with 95% confidence intervals (CIs). Supplementary analyses were conducted using the MR-Egger, weighted median method, simple mode, and weighted mode (19). The IVW method combines SNP-specific instrumental variable (IV) estimates in a meta-analysis framework, weighting each estimate by the inverse of its variance. This method provides a robust estimate of the causal effect when the IV assumptions hold (30). The weighted median method was employed as an additional analysis approach. It generates reliable estimates as long as more than half of the weights are derived from valid instruments (31). The MR-Egger regression was used to adjust for pleiotropy, which refers to the situation where the IVs affect the outcome (HCC) through pathways other than exposure (T2D). However, it is important to note that this method may have reduced statistical power compared to the IVW method (32). The p-value for the MR-Egger intercept and funnel plots were utilized to assess the directional pleiotropy (32). Leave-one-out sensitivity analysis was performed to evaluate the influence of individual SNPs on HCC. Additionally, MR-Pleiotropy residual sum and outlier (MR-PRESSO) was also used to detect outliers (33). To detect heterogeneity within each SNP, the IVW and MR-Egger methods were utilized (32). The extent of heterogeneity among the SNPs was assessed by analyzing the heterogeneity statistic Q (34). Additionally, To further investigate for potential confounding factors(CHB, CHC, BMI and alcohol intake frequency), multivariable MR was used. The reversed MR was conducted to explore the reverse causation. All analyses were conducted in R software (version 4.3.0) using TwoSampleMR and MR-PRESSO packages (33, 35).

## 3 Results

### 3.1 Forward MR analysis

**Figure 2:**
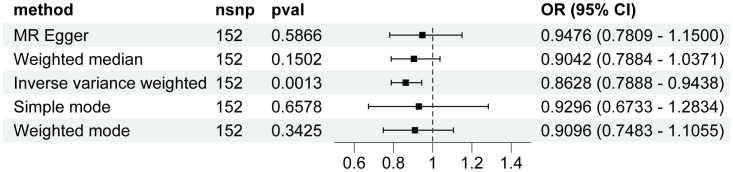
Associations of genetic predisposition to type 2 diabetes with hepatocellular carcinoma. CI, confidence interval; OR, odds ratio; SNPs, single-nucleotide polymorphisms.

**Figure 3:**
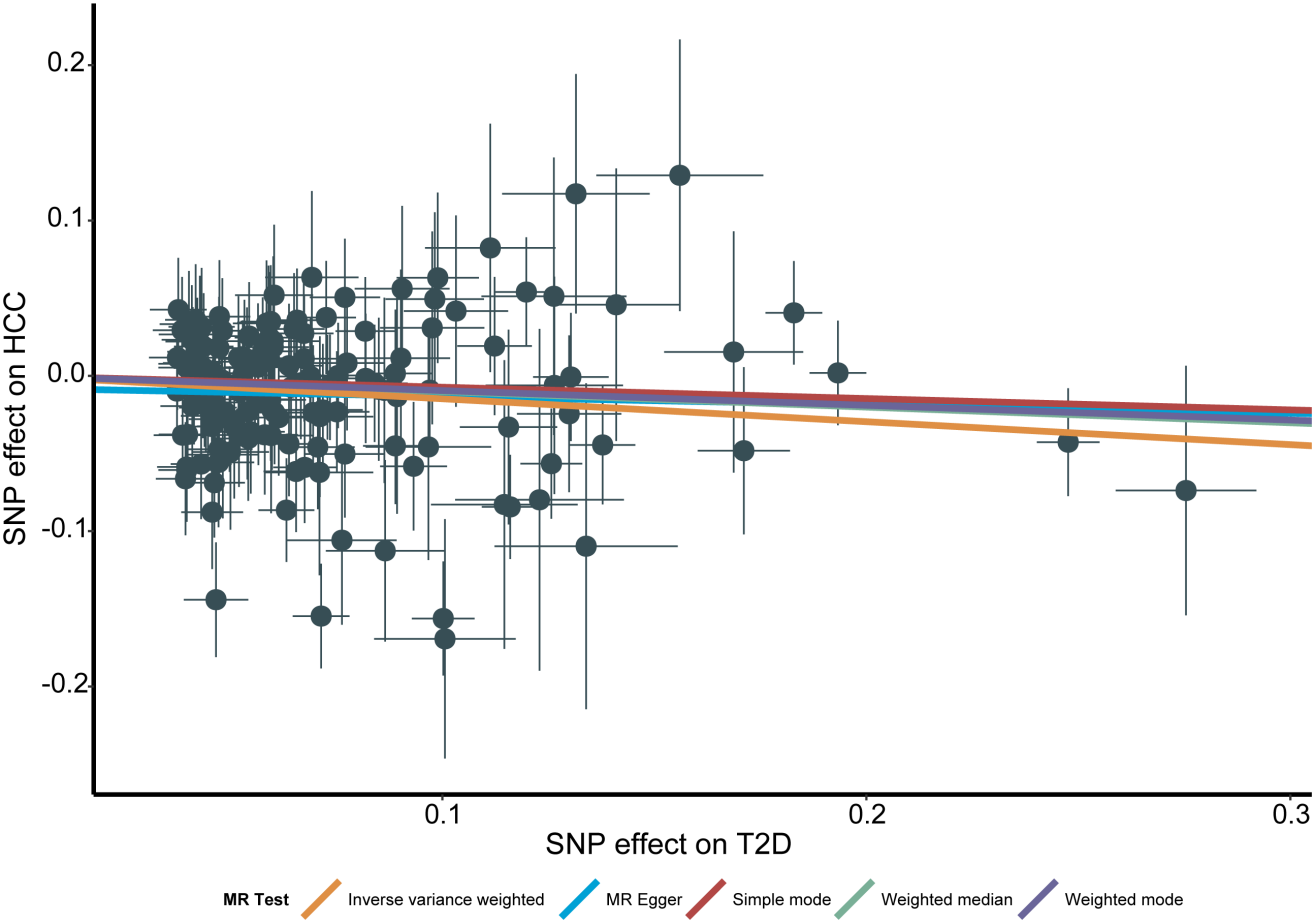
Scatter plot for the associations between type 2 diabetes and the risk of hepatocellular carcinoma. T2D, type 2 diabetes; HCC, hepatocellular carcinoma; MR, Mendelian randomization; SNP, Single nucleotide polymorphism.

**Figure 4:**
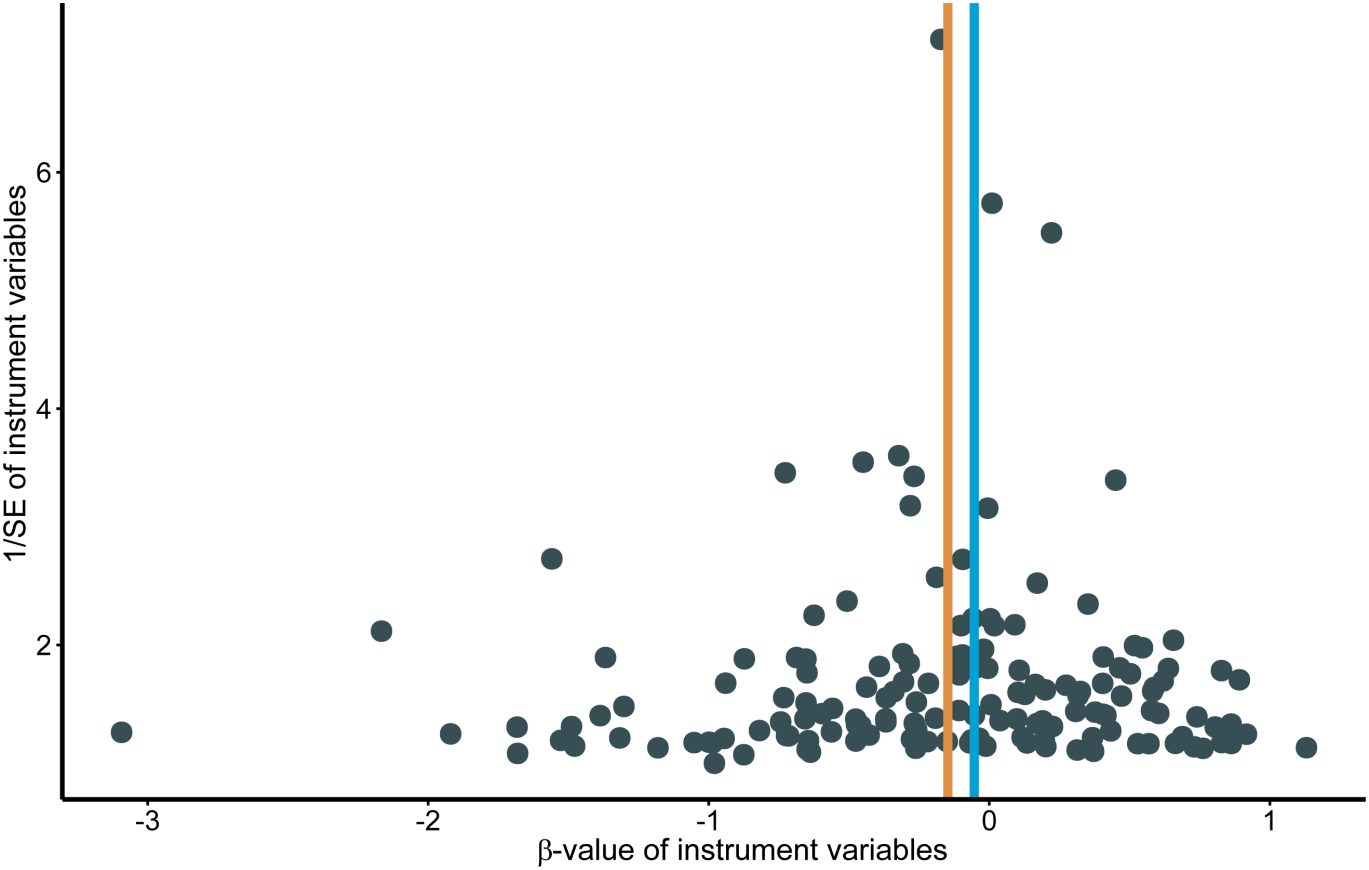
Funnel plots for effects of type 2 diabetes on hepatocellular carcinoma. MR, Mendelian randomization.

**Figure 5:**
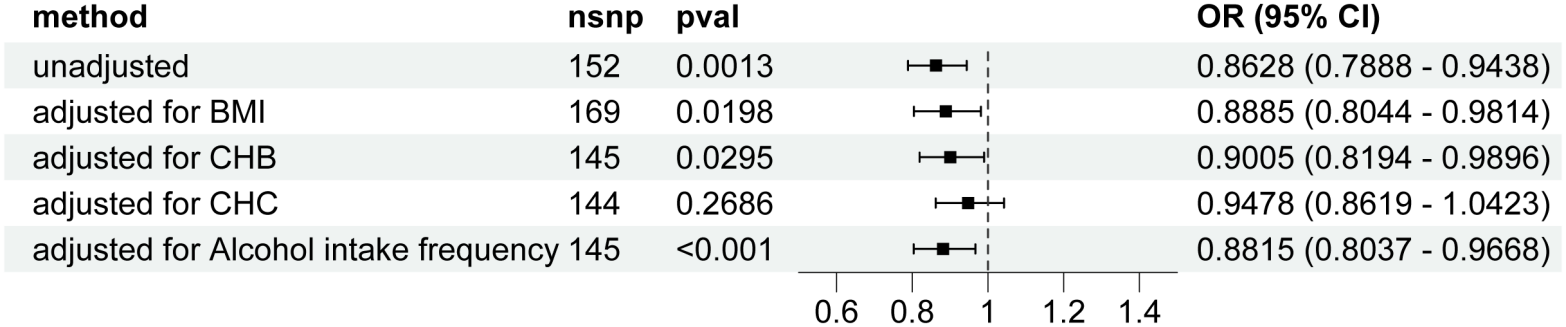
Forest plot for the Mendelian randomization studies investigating the effect of type 2 diabetes on hepatocellular carcinoma using the IVW method after correction for confounders. OR, odds ratio; CI, confidence interval; SNP, Single nucleotide polymorphism; CHB; chronic hepatitis B; CHC, chronic hepatitis C; BMI, body mass index; IVW, inverse variance weighted.

Genetically predicted higher T2D was associated with a decreased risk of HCC (odds ratio per 1-SD increase, 0.8628; 95% confidence interval [CI], 0.7888 to 0.9438; P=.0013). Furthermore, other MR methods also indicated a tendency towards a causal association between T2D and HCC, although not all methods achieved statistical significance (**Figures 2**, **3**). The IVW and MR-Egger methods showed no significant heterogeneity among the SNPs, suggesting that the results are robust (**Table 1**). Additionally, the leave-one-out method demonstrated that no single SNP was driving the observed associations (**Supplementary Figure 1**). MR-PRESSO did not identify any outliers (global test p -value: 0.094). The funnel plots, which are basically symmetrical, and further analysis using the MR Egger regression test, did not provide any evidence of pleiotropy (**Figure 4**). This inverse causality association persisted between T2D and HCC risk after adjustment for a range of potential confounding factors including CHB, BMI and alcohol intake frequency. This trend was still present, although statistically significant differences were not observed after correcting for CHC (**Figure 5**; **Supplementary Tables 3–6**).

**Table 1:** Heterogeneity test among SNPs.

Method	Q	df	Q_pval
Inverse variance weighted	171.346	150	0.112
MR Egger	172.657	151	0.109

SNP, single-nucleotide polymorphisms.

### 3.2 Reverse MR analysis

**Figure 6:**
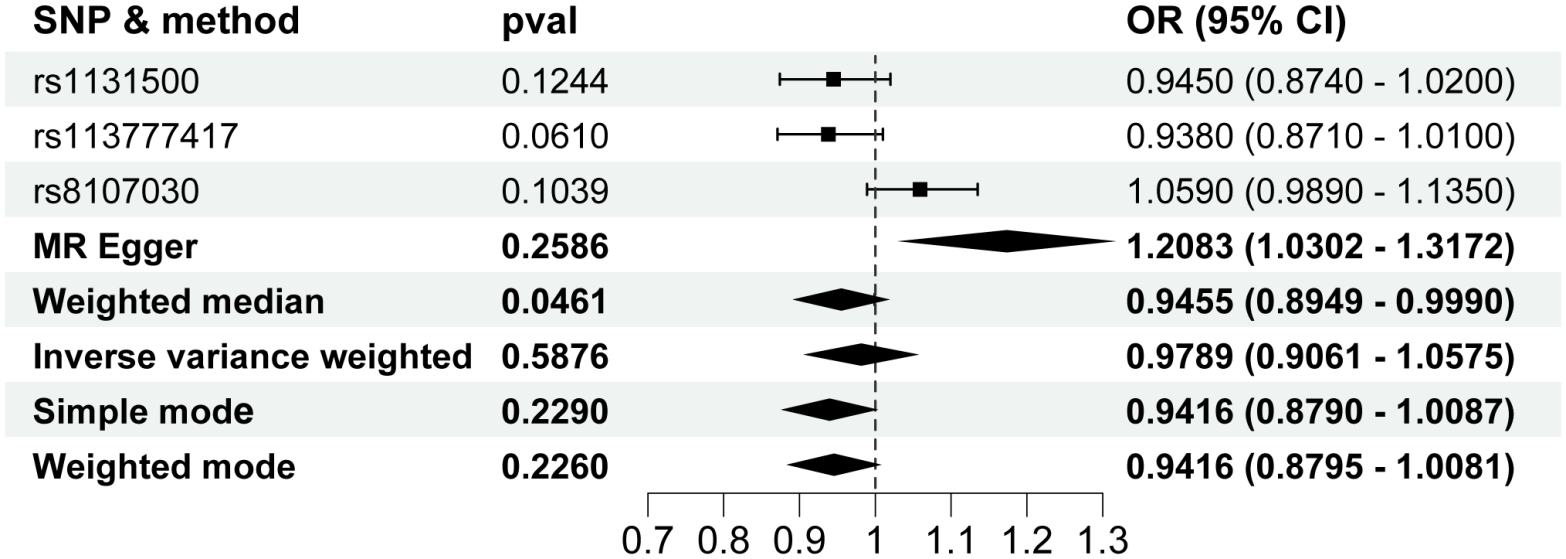
Forest plot for the Mendelian randomization studies investigating the effect of hepatocellular carcinoma on type 2 diabetes. OR, odds ratio; SNP, single-nucleotide polymorphisms.

In the Reverse MR Analysis study, the IVW method did not show a association between HCC and T2D risk. The change of HCC was 0.9788 (95% CI, 0.9061 to 1.0574; P=0.5876) for a 1-unit increase in the log-transformed odds ratio of T2D (**Figure 6**). The inconsistent result was also observed in other MR methods (**Figure 6**). Horizontal pleiotropy was not found using the MR-Egger regression analysis (P=0.2278). The Q-test of heterogeneity by MR Egger was not statistically significant(P=0.6067).

## 4 Discussion

Our MR analysis suggested inverse genetic correlations of T2D with HCC. Forward MR analysis indicated that genetically predicted T2D were robustly associated with a decreased risk of HCC in East Asian descent. However, the data from the reverse MR analysis supporting an impact of HCC on T2D were limited.

The result in our study is inconsistent with the findings of other studies that have shown an association between T2D and a higher risk of liver cancer. For example, In a study of 1.57 million adults enrolled in 14 prospective US-based studies, it was found that T2D was associated with a higher risk of liver cancer. The finding was consistent after correcting for viral hepatitis infection (36). This also accords with a study from Spain, which showed that T2D is a risk factor for HCC development (37). Another study investigates the link between T2D and HCC incidence in African Americans and whether it varies by race. Using data from the Southern Community Cohort Study, researchers followed low-income participants aged 40-79 with and without diabetes. Results indicate that T2D significantly increases the risk of HCC in White/European Americans, but less so in Black/African Americans. Additionally, controlling for T2D lessens the higher risk of HCC among African Americans, suggesting a stronger link between diabetes and liver cancer in White/European Americans. These findings may indicate distinct mechanisms and impacts of T2D on HCC in African Americans versus White/European Americans (38). A possible explanation for this difference with our MR results might be that the subjects in these studies were all European populations. In contrast, our study was limited to East Asian populations. Ethnographic discrepancies may be the main reason for the differences in results.

T2D has been widely reported as an independent risk factor for the risk of HCC (39, 40), and this risk can be reduced with aggressive interventions (41–44). For example, a study conducted in Taiwan found that the use of H1-antihistamines (AHs) was associated with a reduced risk of HCC in T2D patients without hepatitis B virus (HBV) or hepatitis C virus (HCV) infection in a dose-dependent manner (41). Another study found that adherence to a T2D prevention diet, which includes a healthy balance of glycemic index, cereal fibre, polyunsaturated to saturated fats, trans fat, sugar-sweetened beverages, nuts, coffee, and red and processed meats, was associated with a lower risk of HCC among US men and women (42). In patients with both T2D and chronic HCV infection, the use of dipeptidyl peptidase 4 inhibitors (DPP-4 inhibitors), a class of oral antidiabetic drugs, was associated with a lower risk of HCC (43). A two-centre study in a developing country identified several risk factors for HCC in T2D patients, including Chinese and Malay ethnicities interacting with viral hepatitis, weight loss, abdominal pain/discomfort, alcohol consumption, fatty liver, low platelet count, raised alanine transaminase, and alkaline phosphatase levels. The use of statins was found to reduce the risk of HCC by 63% in these patients (44). Our MR study conducted in East Asian populations is consistent with these findings, suggesting that patients with T2D may help reduce this HCC risk. However, it is important to note that the numerous confounding factors may have influenced the interpretation of the results. Our analysis provides more solid evidence than the previous study because our study includes various sensitivity methods to preclude the possibility of bias from horizontal pleiotropy to eliminate causality between T2D and HCC. Furthermore, the causal association persisted after adjustment for potential confounding factors including BMI,CHB and alcohol intake frequency. After adjusting for CHC, the previously observed significant differences were no longer present. This could potentially be attributed to the genetic characteristics of East Asian populations, which have their own unique genetic features and patterns of gene variation. In this population, certain genes may be associated with susceptibility or resistance to HCV infection, but after adjustment, the shared genetic background among East Asians may diminish the strength of this association or mask it entirely.

Our study did not show a clear impact of genetic liability to HCC on reduced T2D risk. Genetic liability to HCC and risk of developing T2D were inconsistent in our MR approach. In addition, there are currently no reports in the literature for HCC and T2D risk.Thus, our inverse MR analysis neither negates nor confirms the effect of HCC on T2D.

Our study has several strengths. First, We conducted a bidirectional MR study to clarify the causal inference between T2D and HCC. This design can significantly reduce the risk of causal inference errors caused by genetic bias and yield more reliable conclusions. Second, The association between T2D and HCC was further verified after adjusting for confounding factors that have been shown to have a close relationship with the development of HCC. The characteristics of our study design make the results particularly robust. In addition, we limited our analysis to East Asian populations. This reduces bias due to differences in population structure.

There are some limitations of this study that should be considered. Firstly, our MR analysis was limited to individuals of East Asian ancestry due to the availability of relevant genetic data. Due to regional and ethnic differences, these results may not be fully applicable to other populations. Secondly, despite controlling for confounding factors such as BMI, CHB, CHC and alcohol intake frequency, there may still be unaccounted confounders that could influence the study results, including age, gender, family history, and chronic liver diseases (such as non-alcoholic fatty liver disease and cirrhosis). Further research could explore additional potential confounding variables. Thirdly, the study relied on existing data resources, and there may be limitations in data acquisition and measurement regarding the specific relationship between T2D and HCC occurrence. Future studies could consider using more accurate and detailed data collection methods. Additionally, while the study utilized MR analysis to infer a inverse association between T2D and HCC risk, further research is needed to validate and confirm causality. Other study designs such as longitudinal studies or experimental research may provide stronger evidence of causality.

In conclusion, Our study, based on GWAS data, suggests that East Asians with genetic susceptibility to T2D are less susceptible to HCC. The findings may have important implications for the prevention and clinical management of these conditions. Whether HCC has a causal effect on reducing T2D warrants more study.

## Data availability statement

The original contributions presented in the study are included in the article/**Supplementary Material**, further inquiries can be directed to the corresponding author/s.

## Author contributions

JH: Conceptualization, Software, Writing – original draft, Writing – review & editing. YX: Data curation, Software, Writing – review & editing. XC: Writing – review & editing. JY: Writing – review & editing. LZ: Funding acquisition, Supervision, Writing – review & editing.
