# Supplementary material for: Inverse association between type 2 diabetes and hepatocellular carcinoma in East Asian populations
Source: Front Endocrinol (Lausanne). 2024 Jan 3;14:1308561. doi: 10.3389/fendo.2023.1308561 (PMC10791969; doi:10.3389/fendo.2023.1308561)
Supplement: Supplementary file 2 [file Image_1.pdf]

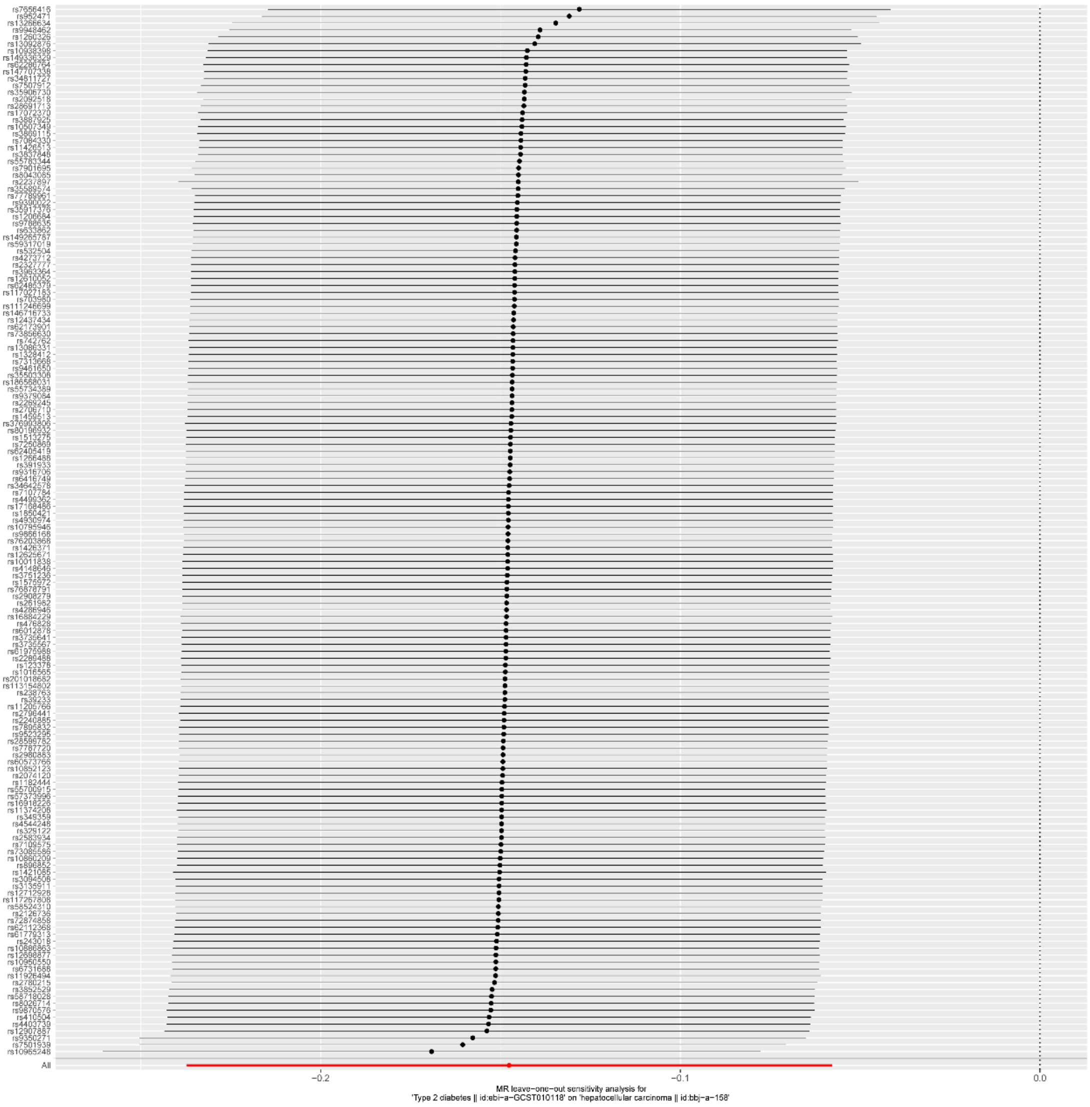

Figure S1 Leave-one-out of SNPs associated with type 2 diabetes（ebi-a-GCST010118） and hepatocellular carcinoma.

SNP , single-nucleotide polymorphism.
